# Supplementary material for: Schiff Bases Derived from Pyridoxal 5′-Phosphate and 2-X-Phenylamine (X = H, OH, SH): Substituent Effects on UV-Vis Spectra and Hydrolysis Kinetics
Source: Molecules. 2024 Jul 26;29(15):3504. doi: 10.3390/molecules29153504 (PMC11314395; doi:10.3390/molecules29153504)
Supplement: Supplementary file 1 [file molecules-29-03504-s001.zip › molecules-3096779-supplementary.pdf]

Supporting Information for

Schiff Bases Derived from Pyridoxal 5′-Phosphate and 2-X-Phenylamine (X = H, OH, SH): Substituent Effects on UV-Vis Spectra and Hydrolysis Kinetics

Maksim N. Zavalishin [1], Aleksei N. Kiselev [2] and George A. Gamov [1,*]

[1] Department of General Chemical Technology, Ivanovo State University of Chemistry and Technology, Sheremetevskii Pr. 7, Ivanovo 153000, Russia; zavalishin00@gmail.com

[2] G.A. Krestov Institute of Solution Chemistry, Russian Academy of Science, Akademicheskaya Str. 1, Ivanovo 153045, Russia; scatol@yandex.ru

[*] Correspondence: ggamov@isuct.ru; Tel.: +7-(915)821-85-62

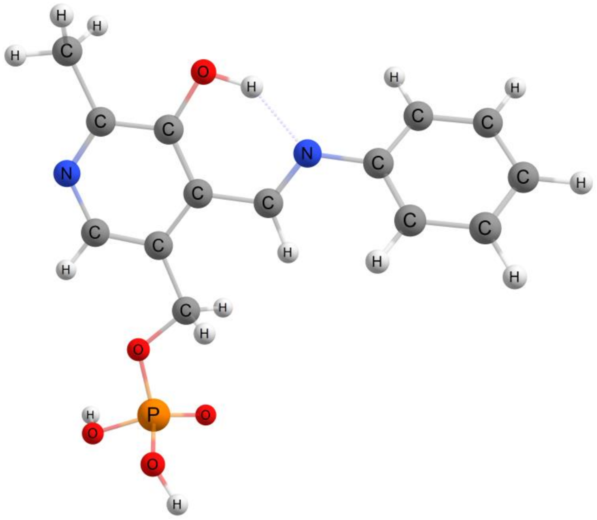

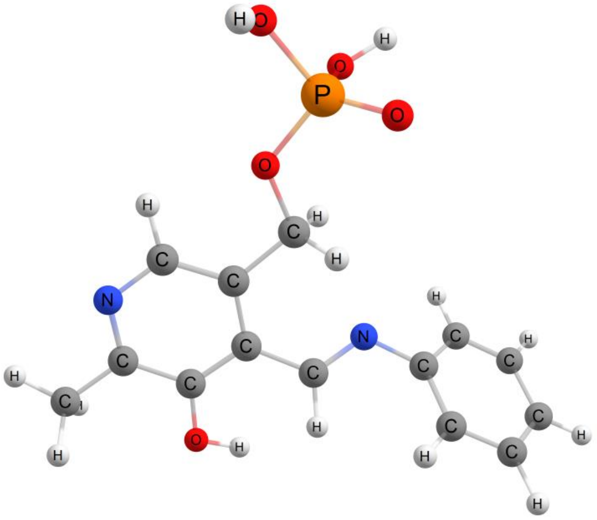

a

b

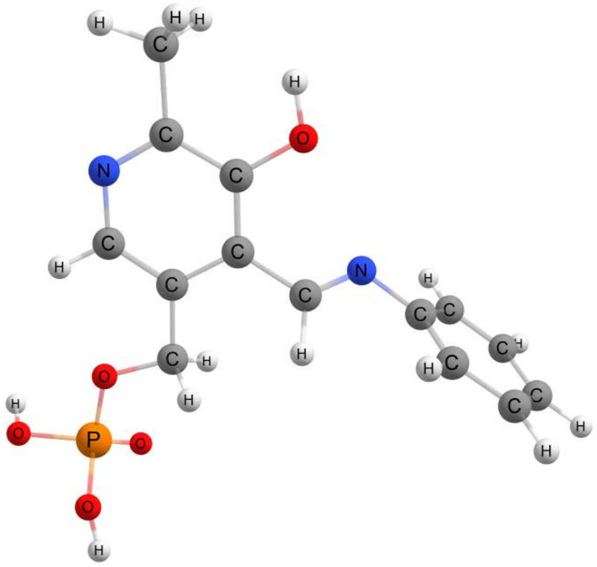

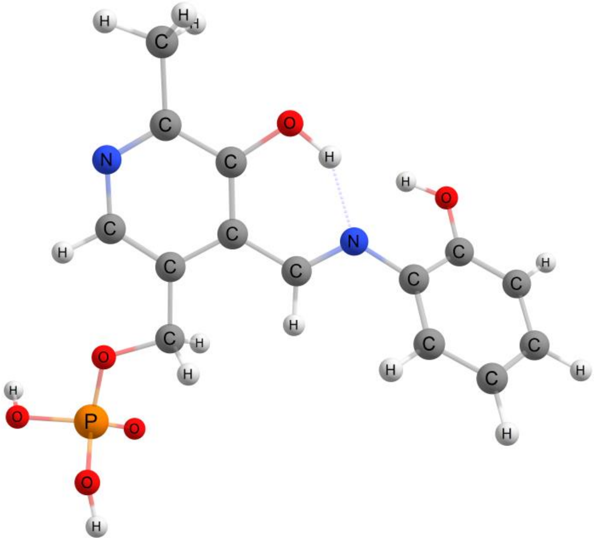

c

d

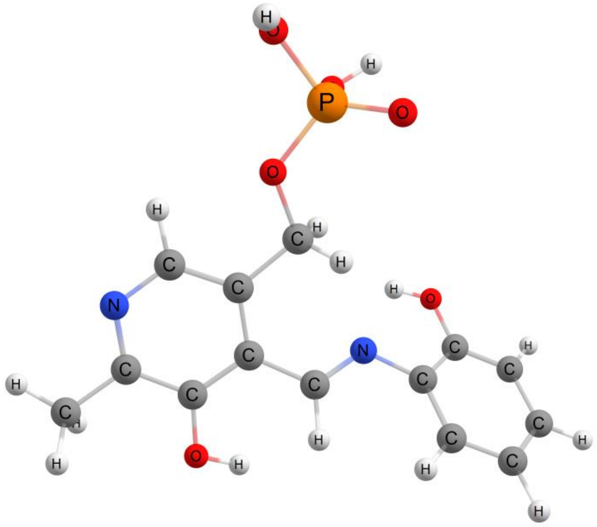

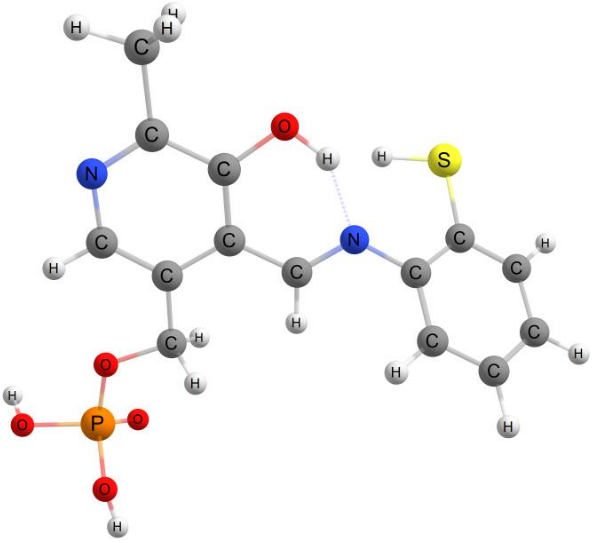

e

f

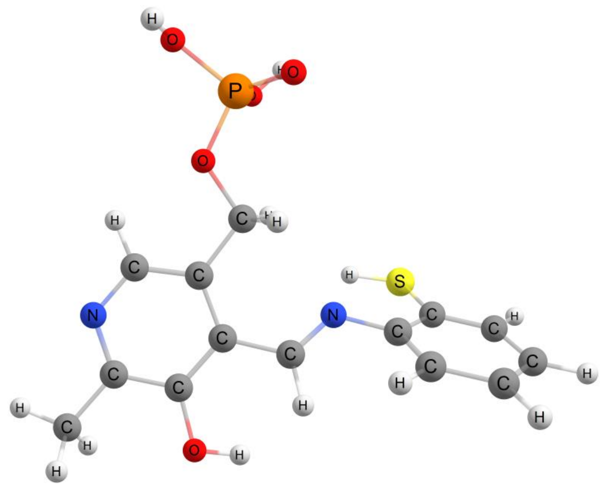

g

Figure S1. Optimized structures of SBs **1** (a-c), **2** (d, e), **3** (f, g). Different rotation conformers are shown: structures b, e, g are obtained from the structures a, d, f, respectively *via* rotation of the pyridoxal 5'-phosphate residue. Conformer c is obtained from the structure a *via* rotation of OH group in position 3 of pyridoxal 5'-phosphate

Table S1. Xyz coordinates of atoms (optimized geometry), calculated IR frequencies and vertical electron transitions of conformers in ground state of Schiff base derived from pyridoxal 5′-phosphate and aniline

Conformer 1 (relative total energy = 0)

6	-1.892050000	2.071434000	0.034874000
6	-1.056321000	0.976652000	0.005669000
6	0.335045000	1.200868000	0.019267000
6	0.784335000	2.525765000	0.059427000
6	-0.151237000	3.579482000	0.086873000
7	-1.449159000	3.337333000	0.074365000
1	-2.964622000	1.936253000	0.026772000
6	0.329135000	4.997404000	0.131058000
1	0.945689000	5.174308000	1.015508000
1	0.948534000	5.228056000	-0.738937000
1	-0.525516000	5.670935000	0.150319000
8	2.083962000	2.846040000	0.072743000
1	2.598995000	1.993368000	0.044645000
6	-1.604934000	-0.424843000	-0.037436000
1	-1.268697000	-0.949559000	-0.934449000
1	-1.284882000	-0.992552000	0.840124000
8	-3.038824000	-0.369371000	-0.048496000
15	-3.910275000	-1.694076000	-0.163046000
8	-5.388497000	-1.112665000	-0.225317000
1	-5.673113000	-0.807751000	-1.096555000
8	-3.837634000	-2.277405000	1.316474000
1	-4.011011000	-3.224450000	1.390376000
8	-3.523845000	-2.626045000	-1.238869000
6	1.290289000	0.095970000	-0.009373000
1	0.900284000	-0.921811000	-0.053419000
7	2.549994000	0.307588000	0.002777000
6	3.466177000	-0.766426000	0.016360000
6	3.242226000	-1.933474000	0.746581000
6	4.652679000	-0.616357000	-0.699683000
6	4.186672000	-2.950445000	0.730265000
1	2.347756000	-2.036033000	1.348857000
6	5.586229000	-1.640957000	-0.720089000
1	4.825175000	0.303315000	-1.245335000
6	5.356198000	-2.811901000	-0.006469000
1	4.010606000	-3.851248000	1.305708000
1	6.500028000	-1.522172000	-1.289587000
1	6.091094000	-3.607442000	-0.014564000

IR frequency, cm^{-1}	IR intensity, rel. units	IR frequency, cm^{-1}	IR intensity, rel. units	IR frequency, cm^{-1}	IR intensity, rel. units
19.4588	0.2693	671.092	12.8544	1317.5008	4.8995
25.314	0.989	716.9701	67.2897	1325.9406	35.1809
31.8693	0.3904	734.8055	11.0715	1344.0922	61.3829
39.6847	0.4067	768.1019	10.1109	1359.1023	3.7586
55.4462	0.1186	786.0192	28.718	1410.5721	49.1889
73.2438	8.7876	802.5699	30.8769	1421.0718	32.4641
85.1824	1.2056	851.9009	71.5567	1427.5115	43.7245
121.8481	3.5131	861.8877	162.9095	1448.3298	374.616
129.3701	10.4802	864.4256	0.6514	1459.2892	2.6435
137.4771	4.8003	878.4698	43.872	1470.9425	14.8478
155.3706	138.5459	893.1181	459.165	1480.0454	40.9068
168.087	5.4898	916.4503	508.08	1499.6165	3.4744
184.911	14.9897	930.4727	10.2823	1508.868	10.8383
191.5185	5.8353	950.6739	14.0472	1535.9086	49.5603
208.9681	5.9169	984.9533	8.899	1567.3144	2.039
281.2734	1.8196	1010.5084	147.0838	1629.3583	29.7422
297.0419	167.7486	1013.387	1.8283	1654.1546	6.3471
299.1558	3.1406	1016.6027	2.6722	1665.1919	84.2526
332.5536	6.9634	1026.7397	5.321	1683.37	9.266
342.3486	4.3518	1030.2815	123.829	1717.7498	224.4439
359.1974	18.7143	1034.7495	16.012	3058.7549	24.2122
365.7544	68.0058	1035.542	61.4171	3063.3877	16.0959
385.0018	56.1152	1041.7188	12.6233	3111.3431	11.389
427.4141	2.3745	1057.8576	23.0297	3112.9598	16.482
449.2873	113.5257	1063.3788	3.4507	3118.1495	266.4576
454.2273	68.6809	1075.3169	366.1005	3140.6934	893.9941
469.1488	35.6708	1113.194	10.2221	3160.7455	5.8403
486.8764	14.5131	1124.072	273.9307	3193.2183	1.6747
527.0936	21.9626	1178.9574	0.3667	3199.2763	2.45
533.1405	18.4146	1197.756	2.879	3207.0058	10.0767
548.854	3.5616	1233.3924	179.2245	3212.8748	18.1947
595.2639	16.1478	1252.8377	59.306	3220.1092	12.1542
597.62	28.0802	1270.0692	528.0751	3227.8944	5.7628
608.0399	6.7651	1300.1208	31.8919	3835.3595	306.9801
636.9685	0.1147	1311.1165	3.5457	3843.0549	213.1882

Wavelength, nm	Oscillator strength, rel. units
354.78	0.3492
333.41	0.0965
309.68	0.165
291.02	0.0082
267.77	0.2151
242.59	0.0115
238.11	0.0297
227.37	0.0022
226.99	0.0214
224.36	0.0062

Conformer 2 (relative total energy = 36.6 kJ mol^{-1})

6	-2.307977000	1.644937000	0.146743000
6	-1.108431000	0.959697000	0.113037000
6	0.080571000	1.713858000	0.037639000
6	-0.039507000	3.104246000	0.018051000
6	-1.305004000	3.709908000	0.049979000
7	-2.405038000	2.977863000	0.113007000
1	-3.238621000	1.096824000	0.194357000
6	-1.426335000	5.202815000	0.017221000
1	-0.906736000	5.656491000	0.864197000
1	-0.976513000	5.614167000	-0.889354000
1	-2.477953000	5.480688000	0.051398000
8	1.012527000	3.961168000	-0.031539000
1	1.854904000	3.520659000	0.115409000
6	-1.092573000	-0.544105000	0.135753000
1	-0.459724000	-0.941880000	-0.657225000
1	-0.712839000	-0.917425000	1.086925000
8	-2.440822000	-1.023761000	-0.047855000
15	-2.746243000	-2.574301000	-0.183507000
8	-4.321022000	-2.585890000	-0.411989000
1	-4.603647000	-2.414562000	-1.319459000
8	-2.621647000	-3.086390000	1.319999000
1	-2.442032000	-4.030489000	1.413577000
8	-1.937991000	-3.309245000	-1.175440000
6	1.415417000	1.107315000	-0.058223000
1	2.207770000	1.722355000	-0.498960000
7	1.667202000	-0.074384000	0.330412000
6	2.958305000	-0.608448000	0.137580000
6	3.678534000	-0.425611000	-1.043616000
6	3.500352000	-1.387411000	1.159888000
6	4.938176000	-0.993089000	-1.182649000
1	3.242497000	0.133306000	-1.862845000
6	4.765387000	-1.936743000	1.020659000
1	2.922731000	-1.543728000	2.062771000
6	5.489178000	-1.742011000	-0.150986000
1	5.487055000	-0.854481000	-2.106440000
1	5.184755000	-2.527740000	1.826006000
1	6.471932000	-2.183233000	-0.263557000

IR frequency, cm^{-1}	IR intensity, rel. units	IR frequency, cm^{-1}	IR intensity, rel. units	IR frequency, cm^{-1}	IR intensity, rel. units
14.1272	1.1263	636.0722	0.877	1296.417	28.1817
26.816	0.7902	640.1442	3.4847	1315.674	11.3467
34.1616	0.2345	718.3746	70.5854	1321.611	34.5937
38.4642	1.541	752.7845	11.061	1329.731	59.4839
50.0073	3.1151	782.4986	6.5077	1357.905	1.951
71.9135	0.3002	787.42	46.9155	1407.717	6.0175
83.944	2.2884	801.1918	23.0241	1415.781	7.1932
118.6326	1.0442	848.2301	58.363	1438.812	160.6176
125.8772	1.1908	865.7402	1.1903	1460.732	58.3578
144.136	42.4531	885.2016	145.052	1471.994	15.5578
147.2603	81.8571	894.5407	462.6284	1477.924	75.5279
160.4793	44.6203	916.812	383.1896	1481.844	23.6555
168.0817	15.7224	930.228	10.9849	1497.801	5.3575
197.5086	14.2122	936.7476	11.938	1537.046	60.887
244.7336	17.751	954.9132	28.9048	1544.078	10.9352
250.3415	5.2907	998.582	36.0269	1632.629	20.9784
269.2343	78.451	1013.211	85.965	1651.846	8.1508
291.8386	147.949	1013.613	28.932	1668.072	33.4295
301.3545	82.6161	1019.703	10.9918	1675.556	44.4102
316.9774	43.1551	1026.506	1.7209	1734.49	167.2777
343.2645	3.7813	1031.941	59.5442	3052.155	48.2835
357.6659	39.262	1032.998	4.9615	3059.985	24.3597
373.2282	59.9915	1036.215	41.6689	3094.867	15.3384
379.0426	34.5409	1058.346	16.0952	3115.153	15.9047
428.1426	2.6329	1061.171	775.8296	3143.192	5.1973
446.6865	93.3022	1065.248	6.555	3160.396	14.6012
453.6039	62.8033	1111.556	24.4672	3191.253	2.1551
464.1564	35.1791	1115.541	47.8672	3197.691	2.4882
481.7733	35.242	1177.912	1.42	3205.399	12.6205
517.9409	33.3969	1196.077	32.6166	3211.655	21.3945
540.7255	9.957	1205.297	273.4611	3218.538	13.4511
543.8982	4.5332	1251.085	91.7242	3228.017	3.9407
590.0534	13.0415	1266.753	501.0296	3837.286	377.9671
606.385	42.2841	1272.165	25.6487	3840.314	133.4297
612.7579	0.3251	1285.992	9.0861	3869.64	161.5414

Wavelength, nm	Oscillator strength, rel. units
347.97	0.3448
324.21	0.076
298.97	0.0472
287.01	0.0085
267.44	0.3267
241.87	0.0372
236.95	0.0466
226.83	0.0127
222.94	0.0072
221.7	0.0097

Conformer 3 (relative total energy = 44.6 kJ mol^{-1})

6	-2.003057000	2.011876000	-0.013438000
6	-1.057225000	1.008811000	0.083694000
6	0.299439000	1.366326000	0.117121000
6	0.612847000	2.723685000	0.072942000
6	-0.417283000	3.674676000	-0.017223000
7	-1.689860000	3.308283000	-0.062180000
1	-3.054908000	1.764249000	-0.054601000
6	-0.096580000	5.141010000	-0.061868000
1	0.402589000	5.475422000	0.853361000
1	0.550650000	5.389567000	-0.908267000
1	-1.020201000	5.706213000	-0.163910000
8	1.909865000	3.105240000	0.142192000
1	1.975979000	4.064551000	0.201563000
6	-1.456056000	-0.442276000	0.125049000
1	-1.013369000	-0.990239000	-0.709712000
1	-1.128449000	-0.909090000	1.057581000
8	-2.885159000	-0.542478000	0.045155000
15	-3.603518000	-1.950669000	-0.124646000
8	-5.131728000	-1.529559000	-0.244566000
1	-5.411337000	-1.247968000	-1.125217000
8	-3.531241000	-2.550009000	1.348434000
1	-3.608404000	-3.511171000	1.401910000
8	-3.074771000	-2.815498000	-1.195900000
6	1.327975000	0.313474000	0.233658000
1	1.070751000	-0.534809000	0.876895000
7	2.432907000	0.345624000	-0.386273000
6	3.362702000	-0.694581000	-0.175424000
6	3.657469000	-1.188721000	1.095757000
6	4.041105000	-1.201934000	-1.283684000
6	4.598312000	-2.199119000	1.247078000
1	3.171251000	-0.763972000	1.965756000
6	4.966256000	-2.222247000	-1.127192000
1	3.824255000	-0.794030000	-2.263517000
6	5.249206000	-2.724786000	0.138719000
1	4.827347000	-2.571195000	2.238611000
1	5.476597000	-2.620811000	-1.995859000
1	5.982287000	-3.512772000	0.260620000

IR frequency, cm^{-1}	IR intensity, rel. units	IR frequency, cm^{-1}	IR intensity, rel. units	IR frequency, cm^{-1}	IR intensity, rel. units
20.4532	0.7276	637.0787	0.322	1310.7959	1.6708
26.2371	0.4894	668.7647	10.4864	1315.6435	5.3552
30.1288	0.5213	717.747	54.7417	1320.6871	33.0891
33.2848	1.0718	730.2894	27.354	1344.5906	0.6663
48.3077	0.4117	765.7407	8.7906	1357.1483	0.7792
71.6804	3.7332	787.9081	34.6666	1414.6779	3.7622
81.4191	1.3111	802.5572	37.4754	1423.5318	32.8218
118.1715	8.4445	850.8489	62.9044	1432.8128	32.8083
125.1016	6.0367	864.9493	16.2482	1459.4463	242.6269
138.6921	13.2046	866.7051	29.0752	1486.1971	20.7697
151.7223	100.1847	892.87	457.9972	1492.0987	51.2624
170.8219	23.3229	915.8766	517.2062	1496.8595	6.0316
178.4331	24.6801	935.5992	14.6906	1507.299	12.8842
198.2229	2.3019	946.1564	23.5326	1533.8991	46.1708
216.3819	21.7995	977.0074	2.1518	1544.9108	14.1279
265.6754	7.261	998.3191	99.8891	1633.9579	9.0953
297.0501	165.1344	1012.5	71.3565	1651.1741	7.2144
303.8264	113.8543	1013.8248	49.5234	1668.4417	52.717
308.7395	24.9711	1022.2164	46.5401	1680.766	3.0558
330.5393	4.2255	1028.6885	69.7358	1744.5527	181.4156
346.9342	40.5186	1032.3532	3.4783	3040.8	19.5723
359.3444	32.1206	1033.7555	84.6691	3060.5162	27.7561
370.4421	4.6524	1041.8586	3.698	3066.3845	42.1133
379.3589	116.7139	1057.8337	18.1917	3093.3619	14.7378
427.118	1.6194	1063.8705	7.4596	3110.6157	7.0699
442.617	87.7164	1068.2118	243.5244	3163.0318	6.9337
453.9623	69.0434	1111.6856	16.9811	3190.3564	2.3902
455.7762	62.0527	1124.1816	237.5356	3196.6155	2.5604
476.548	26.9312	1177.8764	0.5566	3204.7335	12.796
522.0077	46.732	1196.3252	4.1519	3210.4448	23.0342
529.1798	5.1163	1239.9021	75.4176	3217.7233	13.5438
550.8803	2.1812	1245.6884	17.9953	3226.1081	4.5954
588.8431	7.445	1270.3348	513.2369	3834.1451	293.2875
601.8553	13.4121	1275.4732	365.3645	3844.3909	234.2998
606.8985	2.9992	1288.709	0.7843	3847.3409	196.4547

Wavelength, nm	Oscillator strength, rel. units
342.1	0.2732
309.39	0.078
287.22	0.0472
278	0.0063
258.1	0.3088
242.8	0.0496
237.85	0.0486
230.01	0.0022
225.5	0.0442
223.32	0.0743

Table S2. Structure, xyz coordinates of atoms (optimized geometry), calculated IR frequencies and vertical electron transitions of conformers in ground state of Schiff base derived from pyridoxal 5′-phosphate and 2-hydroxyaniline

Conformer 1 (relative total energy = 0)

6	2.099278000	2.063336000	-0.106537000
6	1.262955000	0.969078000	-0.088095000
6	-0.128784000	1.194578000	-0.118017000
6	-0.574637000	2.519540000	-0.164656000
6	0.360989000	3.572740000	-0.189538000
7	1.658093000	3.329320000	-0.157728000
1	3.171428000	1.927440000	-0.080697000
6	-0.117797000	4.990519000	-0.249260000
1	-0.729449000	5.159670000	-1.138529000
1	-0.741184000	5.229847000	0.615507000
1	0.737812000	5.662676000	-0.270171000
8	-1.873899000	2.845248000	-0.195235000
1	-2.393996000	2.005037000	-0.161999000
6	1.811445000	-0.432026000	-0.030388000
1	1.456963000	-0.953180000	0.861565000
1	1.510459000	-1.003470000	-0.912277000
8	3.244371000	-0.375470000	0.006589000
15	4.113762000	-1.684610000	0.248989000
8	5.588346000	-1.094603000	0.315044000
1	5.843781000	-0.722982000	1.169297000
8	4.091260000	-2.378615000	-1.183395000
1	4.278113000	-3.326019000	-1.180158000
8	3.692413000	-2.532514000	1.379680000
6	-1.077925000	0.086039000	-0.123681000
1	-0.679976000	-0.926564000	-0.200526000
7	-2.341819000	0.272442000	-0.051111000
6	-3.230563000	-0.817819000	-0.150415000
6	-3.051030000	-1.879558000	-1.034665000
6	-4.378532000	-0.779835000	0.651088000
6	-3.986671000	-2.899688000	-1.109304000
1	-2.188166000	-1.884204000	-1.689713000
6	-5.312881000	-1.803065000	0.578953000
6	-5.115255000	-2.859088000	-0.298001000
1	-3.842374000	-3.714311000	-1.807389000
1	-6.187606000	-1.757325000	1.215741000
1	-5.852201000	-3.650965000	-0.352724000
8	-4.593667000	0.236270000	1.525191000

1 -3.858097000 0.860135000 1.471227000

IR frequency, cm^{-1}	IR intensity, rel. units	IR frequency, cm^{-1}	IR intensity, rel. units	IR frequency, cm^{-1}	IR intensity, rel. units
21.8984	0.9859	607.6162	7.475	1312.3399	0.9852
29.6759	0.5413	610.1315	8.5407	1323.1538	44.5191
37.2955	0.1937	665.4329	9.7709	1327.4271	61.8915
40.4136	0.3506	728.1177	5.4372	1343.651	53.4871
54.1729	0.8645	764.3597	1.0626	1368.0145	16.6243
75.3898	6.8418	770.2291	22.5868	1408.5396	108.8345
84.3303	2.248	777.4657	102.1348	1419.999	42.3706
121.209	2.4542	795.8409	1.4932	1430.5634	99.9147
130.5222	10.7417	822.8697	137.4766	1441.049	186.9121
136.2591	3.0807	837.9935	61.5915	1457.8647	70.0397
152.2172	26.9353	851.8867	69.119	1471.1862	14.9492
155.9956	96.5147	886.1006	18.5933	1479.7786	61.6932
176.0585	42.0819	893.8331	281.2835	1509.1634	14.8734
190.084	0.4494	894.1854	183.6856	1514.3872	8.1515
207.1411	4.3486	916.5162	514.352	1535.5248	152.8989
229.6088	2.4231	931.8204	12.5018	1562.2555	12.2811
279.2748	5.5475	978.8783	7.0669	1628.2536	32.062
298.1774	164.9813	985.97	9.4333	1657.347	69.0104
305.4746	7.212	1013.624	82.7407	1672.7411	31.6712
318.2123	9.976	1017.1532	81.6151	1685.5808	19.3137
342.4305	5.42	1018.5632	0.5598	1710.1472	239.9958
357.9422	34.6958	1030.1877	117.6334	3059.2852	22.3346
368.0822	9.4404	1037.4668	62.024	3062.9409	19.7391
376.301	95.2004	1042.7854	25.9122	3111.8589	7.1849
426.8215	33.7169	1063.8046	3.1215	3113.7728	15.8792
448.5546	137.845	1068.7372	73.5006	3122.5985	37.4586
450.192	146.2804	1073.5039	317.9239	3161.4612	16.8425
456	70.6137	1123.8031	271.6992	3197.2066	0.0979
469.6173	41.9199	1130.3007	20.0294	3203.7479	4.5722
484.4708	7.0128	1176.1713	36.3801	3213.4238	11.9814
506.3926	7.0234	1201.6458	127.399	3221.8101	15.0868
530.9203	30.2973	1232.0919	119.4844	3228.7993	5.2201
555.6274	6.9443	1249.7892	217.8708	3295.0178	931.9492
563.0232	12.7541	1270.1754	522.6908	3804.8149	200.989
580.066	10.758	1289.3379	116.2849	3836.3476	357.9472
601.6726	14.7599	1304.8773	38.3264	3840.2402	161.6929

Wavelength, nm	Oscillator strength, rel. units
387.48	0.3186
345.96	0.0928
323.26	0.0196
311.15	0.1442
264.14	0.2562
248.42	0.0201
241	0.0135
231.59	0.0122

Conformer 2 (relative total energy = 29.6 kJ mol^{-1})

6	-2.626064000	1.391440000	0.165003000
6	-1.347739000	0.877762000	0.054382000
6	-0.277020000	1.789333000	-0.034609000
6	-0.583174000	3.150098000	0.011859000
6	-1.914841000	3.576392000	0.121731000
7	-2.902741000	2.698677000	0.195014000
1	-3.471537000	0.719847000	0.222588000
6	-2.236774000	5.038830000	0.164215000
1	-1.745639000	5.522503000	1.011603000
1	-1.886920000	5.545375000	-0.738167000
1	-3.313510000	5.169507000	0.252069000
8	0.346025000	4.138797000	-0.041450000
1	1.241274000	3.810273000	0.086718000
6	-1.128679000	-0.609446000	0.009806000
1	-0.432083000	-0.882849000	-0.782199000
1	-0.725379000	-0.971004000	0.956320000
8	-2.394162000	-1.257551000	-0.229902000
15	-2.498178000	-2.841161000	-0.267175000
8	-4.051708000	-3.068673000	-0.522557000
1	-4.320936000	-3.046109000	-1.449663000
8	-2.344687000	-3.227076000	1.270829000
1	-2.046114000	-4.130410000	1.435418000
8	-1.579407000	-3.530874000	-1.192903000
6	1.120845000	1.381413000	-0.220559000
1	1.767823000	2.086962000	-0.751209000
7	1.578492000	0.267743000	0.186847000
6	2.908412000	-0.109492000	-0.074216000
6	3.664012000	0.286798000	-1.176618000
6	3.464058000	-1.006679000	0.849408000
6	4.960260000	-0.175751000	-1.340953000
1	3.224420000	0.935407000	-1.924643000
6	4.763680000	-1.465579000	0.689224000
6	5.508145000	-1.045746000	-0.402855000
1	5.536899000	0.129732000	-2.204727000
1	5.174217000	-2.150389000	1.420770000
1	6.520284000	-1.410424000	-0.529110000
8	2.738048000	-1.420531000	1.916102000
1	1.856027000	-1.024923000	1.832956000

IR frequency, cm^{-1}	IR intensity, rel. units	IR frequency, cm^{-1}	IR intensity, rel. units	IR frequency, cm^{-1}	IR intensity, rel. units
17.9465	0.7314	595.9249	8.4345	1294.529	11.8782
25.4001	2.2768	609.7688	13.046	1301.1295	24.533
30.5787	0.4272	614.7725	7.2318	1319.4882	82.7674
42.3093	0.6696	638.8522	3.5891	1326.931	19.6236
46.9766	4.8827	747.8827	6.8876	1329.6634	79.544
67.1546	0.9399	767.2221	2.4732	1377.3546	33.1257
92.8579	5.924	774.9352	120.0755	1410.8241	23.5652
116.5777	1.8293	779.2275	16.9122	1416.7795	4.3326
123.9403	2.3748	793.0453	6.1652	1436.5761	154.3922
132.8582	6.0967	844.5809	18.561	1460.2465	66.2556
145.0465	19.4837	853.9944	111.743	1472.2041	15.7759
158.0275	70.8284	884.2945	20.2132	1479.2075	92.4088
176.3269	70.1614	892.2066	395.805	1486.5124	7.0734
207.8484	21.7767	899.8825	125.7708	1517.2585	15.9838
211.3131	18.6975	916.8675	403.28	1534.352	177.6147
235.6574	15.6503	935.693	17.5899	1545.3703	9.8386
252.5959	15.1759	939.2409	12.776	1632.126	16.1752
273.0258	130.3169	979.3988	11.4243	1657.2992	67.1972
287.4402	138.934	998.9951	43.4387	1675.0005	32.6586
295.3987	26.3797	1013.7514	116.9662	1679.9484	6.6752
320.7147	32.4666	1017.2463	0.2744	1727.9701	131.2538
342.8843	5.5703	1029.0656	7.2184	3060.6978	21.553
360.238	37.365	1033.0005	73.1909	3069.3917	40.2781
365.3168	37.7879	1041.1334	28.1696	3092.8787	16.9414
381.9734	69.2234	1062.791	742.4553	3115.7853	15.475
409.5038	12.2772	1065.3796	10.5427	3142.5346	7.0077
448.6774	103.3096	1067.7306	27.8024	3162.0287	14.3048
455.183	76.3857	1116.2988	50.5419	3196.3312	0.0616
474.4181	8.4823	1132.2301	6.0052	3203.9431	6.0136
484.7016	51.6956	1175.6457	58.3469	3212.9777	13.7243
493.6018	25.7599	1201.3322	269.5097	3220.8171	13.3978
530.5663	8.5419	1219.5442	109.2015	3226.8608	4.1124
552.0565	164.9153	1253.0772	207.2143	3727.5538	266.2884
556.6816	23.6785	1267.8188	522.3175	3837.8221	358.4605
570.792	2.9546	1279.3689	43.241	3841.1333	154.2773
575.5685	17.5971	1288.5196	128.2649	3864.2297	170.0849

Wavelength, nm	Oscillator strength, rel. units
380.57	0.3921
336.3	0.083
315.36	0.0686
304.73	0.0806
273.08	0.1957
251.38	0.0365
241.22	0.0034
238.52	0.0259

Table S3. Structure, xyz coordinates of atoms (optimized geometry), calculated IR frequencies and vertical electron transitions of conformers in ground state of Schiff base derived from pyridoxal 5′-phosphate and 2-mercaptoaniline

Conformer 1 (relative total energy = 0)

6	2.266588000	2.082057000	-0.127429000
6	1.455277000	0.969021000	-0.113657000
6	0.061627000	1.160653000	-0.207460000
6	-0.413142000	2.472901000	-0.308482000
6	0.499007000	3.547249000	-0.319307000
7	1.798787000	3.335571000	-0.228397000
1	3.339668000	1.972538000	-0.055869000
6	-0.009153000	4.951541000	-0.432231000
1	-0.588158000	5.085644000	-1.348888000
1	-0.672492000	5.195569000	0.400946000
1	0.830702000	5.643614000	-0.434825000
8	-1.716647000	2.765588000	-0.399798000
1	-2.218321000	1.909619000	-0.373599000
6	2.032591000	-0.416877000	0.000721000
1	1.659511000	-0.922883000	0.893799000
1	1.775031000	-1.017806000	-0.875131000
8	3.461936000	-0.326303000	0.085514000
15	4.352273000	-1.619295000	0.338829000
8	5.815144000	-1.002678000	0.419452000
1	6.060412000	-0.639529000	1.280239000
8	4.354769000	-2.314035000	-1.093475000
1	4.556268000	-3.258406000	-1.088779000
8	3.936207000	-2.474114000	1.466288000
6	-0.865606000	0.032750000	-0.213232000
1	-0.449870000	-0.975626000	-0.189750000
7	-2.131934000	0.205966000	-0.245061000
6	-3.001246000	-0.899000000	-0.339808000
6	-2.749769000	-1.941036000	-1.232935000
6	-4.171323000	-0.922310000	0.431502000
6	-3.637252000	-2.997081000	-1.360803000
1	-1.865321000	-1.897318000	-1.856687000
6	-5.051199000	-1.993246000	0.302959000
6	-4.791683000	-3.021882000	-0.589545000
1	-3.431560000	-3.791099000	-2.067537000
1	-5.947150000	-2.021115000	0.911722000
1	-5.493995000	-3.841090000	-0.680935000
1	-3.405014000	0.958987000	1.635102000
16	-4.603834000	0.360090000	1.582272000

IR frequency, cm^{-1}	IR intensity, rel. units	IR frequency, cm^{-1}	IR intensity, rel. units	IR frequency, cm^{-1}	IR intensity, rel. units
18.2446	1.1148	606.1617	15.3661	1308.7314	7.1551
26.8607	0.9254	609.0053	7.2231	1311.4754	2.9808
37.2132	0.6621	663.5019	7.6863	1315.5348	4.3372
39.788	0.237	705.6211	13.5237	1325.7764	41.1919
51.6229	0.5752	737.5128	14.4232	1343.8462	58.7291
73.4019	7.6651	758.6695	42.3599	1410.1549	81.2357
82.2274	4.4652	767.9336	22.4287	1420.4928	34.1046
107.3092	14.0719	785.9615	40.5997	1431.4814	106.93
127.1937	7.337	798.6523	12.5935	1443.8819	192.0423
132.4276	19.3912	843.9946	162.1519	1457.0476	59.0677
137.3037	5.0077	852.0634	70.2624	1471.8487	15.0806
139.9603	6.5973	876.946	41.034	1479.7829	54.655
156.6707	114.9231	893.8635	461.9378	1486.5931	20.9636
172.6533	43.6514	896.1297	2.2822	1512.0179	12.6451
187.8374	3.4064	916.8655	513.341	1523.4073	61.1421
200.8836	5.3182	924.6889	1.3839	1563.3002	3.0349
209.561	6.4173	930.4127	11.8502	1628.7374	38.159
260.1227	8.8671	983.4656	4.4692	1642.3835	10.0618
295.3178	163.6682	988.5285	8.1249	1657.5231	41.6171
301.9235	4.6318	1015.6841	166.2552	1679.7381	36.6532
312.0007	5.3726	1020.3941	2.3765	1713.9741	281.7778
340.2321	5.3254	1025.1796	0.3323	2745.8796	13.1196
344.5611	8.092	1030.1212	117.8763	3059.2594	22.7196
362.9097	32.4944	1037.9631	72.0569	3062.3417	19.0897
374.3904	83.9097	1044.8301	13.4039	3110.7403	7.6947
395.7957	37.5712	1064.1012	2.7837	3113.6663	16.2442
413.4363	7.3618	1073.7563	350.9057	3120.3072	50.1483
449.6215	107.5805	1076.6597	28.4921	3161.0253	19.9916
456.2034	57.9504	1104.5663	113.4441	3194.7706	3.0007
469.2888	11.7329	1124.798	226.463	3201.4202	1.2428
476.271	40.1839	1165.8856	6.017	3209.9392	7.8422
512.1257	9.0971	1187.0817	1.1671	3217.5287	1050.3269
533.2676	26.4621	1233.2474	190.6791	3221.3344	13.7814
543.4903	8.6689	1252.4912	49.2736	3228.598	12.4182
565.5188	4.1382	1270.1077	525.0207	3836.6728	366.685
599.8177	16.1462	1299.6489	50.0616	3840.2097	154.049

Wavelength, nm	Oscillator strength, rel. units
333	0.315
305.31	0.1248
282.78	0.0235
268.36	0.1461
247.81	0.2339
239.27	0.1967
223.89	0.0162
219.84	0.0848

Conformer 2 (relative total energy = 32.5 kJ mol^{-1})

6	-2.801127000	1.322021000	0.165750000
6	-1.510733000	0.854517000	0.005602000
6	-0.473544000	1.804084000	-0.088886000
6	-0.821314000	3.152070000	0.003566000
6	-2.163481000	3.530395000	0.160799000
7	-3.118953000	2.618646000	0.237897000
1	-3.622438000	0.621856000	0.229980000
6	-2.532180000	4.979618000	0.249670000
1	-2.034370000	5.458488000	1.095871000
1	-2.224020000	5.519890000	-0.648368000
1	-3.609851000	5.071552000	0.369387000
8	0.071016000	4.173762000	-0.046931000
1	0.981828000	3.874148000	0.033182000
6	-1.242656000	-0.622750000	-0.085928000
1	-0.588469000	-0.853690000	-0.926287000
1	-0.764849000	-0.988806000	0.823205000
8	-2.497428000	-1.310620000	-0.263892000
15	-2.552572000	-2.896321000	-0.306886000
8	-4.102733000	-3.171416000	-0.535303000
1	-4.394489000	-3.127474000	-1.454878000
8	-2.361569000	-3.286740000	1.225751000
1	-2.030917000	-4.180887000	1.378989000
8	-1.628093000	-3.551710000	-1.251725000
6	0.930771000	1.443636000	-0.322020000
1	1.554670000	2.191851000	-0.822724000
7	1.421959000	0.321528000	0.013224000
6	2.753648000	0.013985000	-0.321699000
6	3.288445000	0.312921000	-1.575582000
6	3.542950000	-0.673426000	0.613218000
6	4.590971000	-0.035992000	-1.895204000
1	2.660718000	0.796684000	-2.314169000
6	4.852302000	-1.012346000	0.286118000
6	5.375986000	-0.695156000	-0.958282000
1	4.985968000	0.196543000	-2.876206000
1	5.465379000	-1.533651000	1.011935000
1	6.394370000	-0.975444000	-1.197466000
1	1.687012000	-0.671582000	2.029036000
16	2.944828000	-1.100222000	2.229252000

IR frequency, cm^{-1}	IR intensity, rel. units	IR frequency, cm^{-1}	IR intensity, rel. units	IR frequency, cm^{-1}	IR intensity, rel. units
14.2163	1.4829	593.2764	11.1929	1293.1589	14.5206
22.4079	0.3202	609.4646	24.9461	1297.8044	39.6387
32.6015	0.8025	614.2604	2.5457	1307.8848	9.6011
41.1821	5.8762	639.3825	2.5247	1313.4218	5.4071
43.1832	1.4581	702.319	15.7737	1320.9593	43.9961
66.3955	0.6555	750.1621	7.0633	1330.22	59.598
85.6212	2.0475	757.9426	42.18	1410.932	22.2892
108.2339	3.5145	779.981	48.4265	1416.5724	4.318
125.7964	0.1124	784.5561	33.6089	1436.4142	119.7358
127.79	7.0817	802.5779	12.4179	1459.0004	89.6362
144.5701	17.7993	849.0532	60.647	1472.2589	16.1403
155.893	67.9131	882.1831	81.5073	1478.4615	91.1927
163.5493	5.1742	892.9082	410.2785	1483.4022	11.4391
171.9704	80.2871	899.981	109.2522	1486.1326	20.4795
200.5323	10.3966	909.6159	5.8179	1522.5145	71.8432
214.6325	9.5601	917.2666	391.1437	1544.4542	15.3895
239.344	136.1947	935.2624	19.393	1632.7739	24.0239
252.7629	19.1301	939.3252	15.708	1640.8991	3.6886
254.8037	59.1942	984.4322	10.2073	1656.6426	30.1737
287.8921	12.7209	998.9718	42.7073	1675.4598	27.1513
291.726	153.6478	1013.0185	111.6266	1731.7172	209.4373
315.5792	22.9906	1023.0581	2.9012	2719.9244	37.7958
326.1318	14.4937	1025.0146	7.5147	3059.459	33.5952
343.7748	2.4282	1033.0198	69.5838	3060.5936	30.7726
361.7082	41.5586	1036.4905	42.1954	3093.8826	16.5262
378.4218	98.7894	1062.3911	743.5992	3115.8112	15.3196
384.5268	1.0236	1065.3453	8.3935	3142.3432	6.5291
411.5388	3.823	1075.4976	26.5852	3161.5375	13.8928
448.1042	100.5611	1103.6549	41.7358	3193.0497	2.4062
455.7024	74.6797	1116.7886	56.6225	3199.6203	2.0357
469.7322	3.6317	1164.2356	11.9039	3208.2749	12.2331
480.0623	35.4815	1184.8738	1.6282	3219.6344	16.1667
507.0831	24.6507	1208.2806	311.6815	3227.9265	3.7666
528.0843	12.9893	1249.4295	74.525	3838.9501	365.1751
553.6583	22.1425	1267.5223	522.1866	3842.2636	147.6838
558.5381	6.3197	1277.9806	15.2173	3865.8583	168.0188

Wavelength, nm	Oscillator strength, rel. units
332.7	0.2737
294.26	0.1598
278.23	0.0218
263.48	0.0764
248.65	0.2718
239.98	0.2988
225.55	0.0434
222.07	0.0519

Table S4. Molecular orbitals involved in electron transitions of the Schiff bases derived from pyridoxal 5′-phopshate and aniline (**1**), 2-hydroxyaniline (**2**), and 2-mercaptoaniline (**3**)

Schiff base/conformer	Orbitals
**1**/1	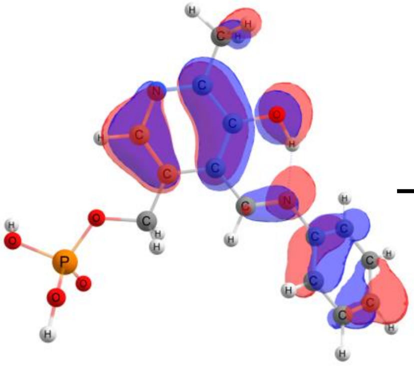 HOMO 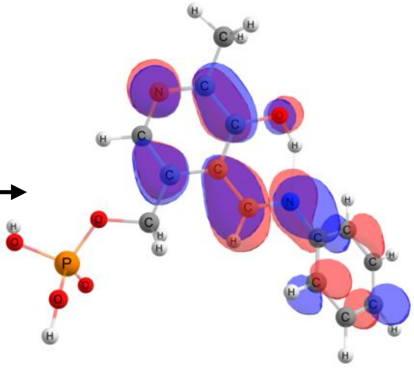 LUMO<br>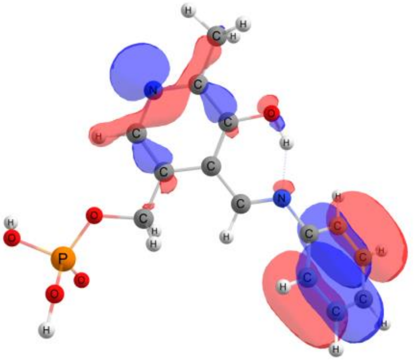 HOMO-3<br>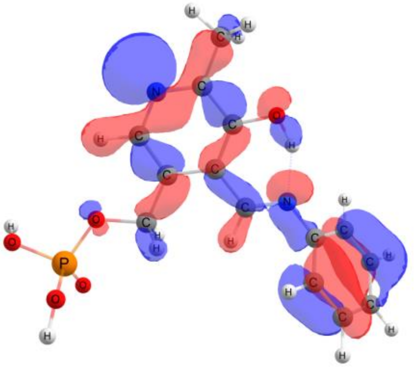 HOMO-2 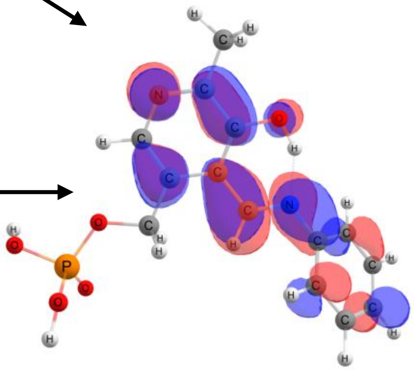 LUMO<br>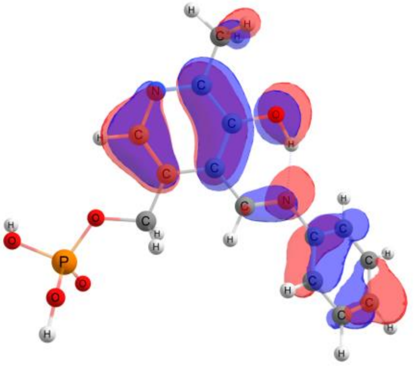 HOMO-1

	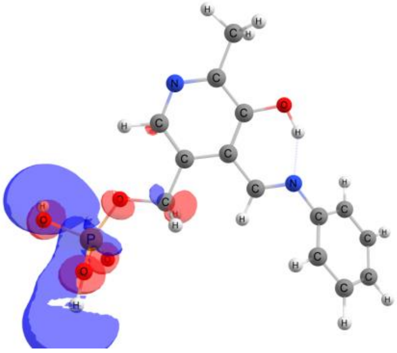<br>LUMO+2
**1**/2	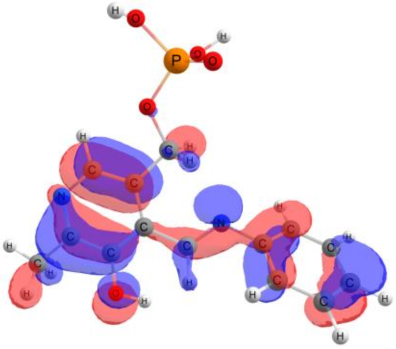 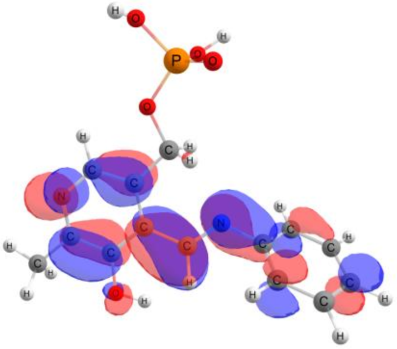<br>HOMO-1 LUMO<br>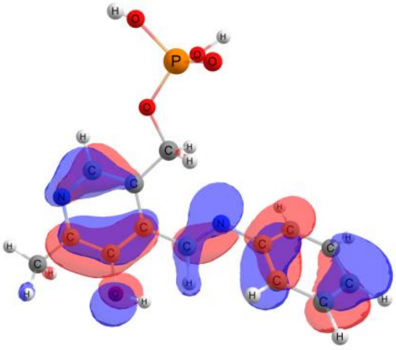<br>HOMO<br>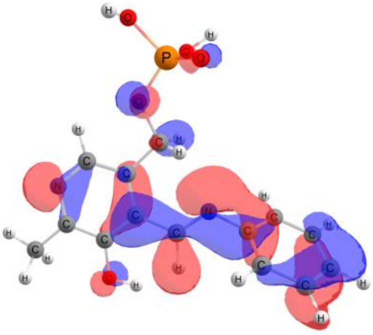 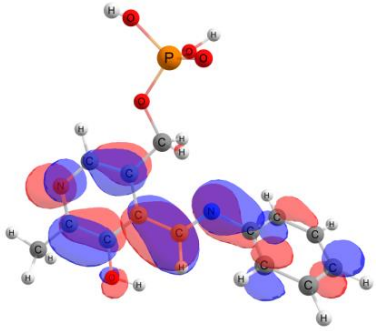<br>HOMO-5 LUMO<br>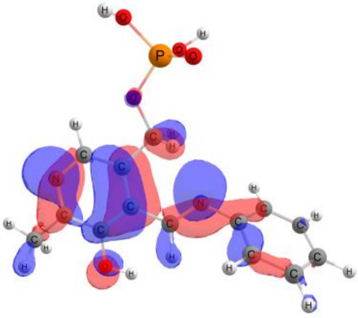<br>HOMO-4

	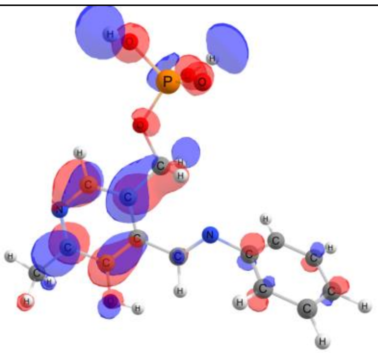<br>LUMO+2
**1**/3	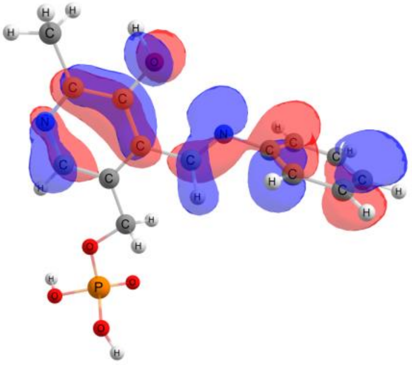 HOMO → 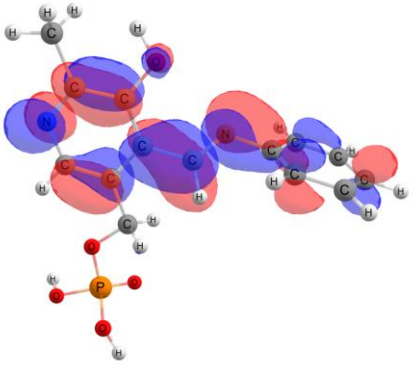 LUMO<br>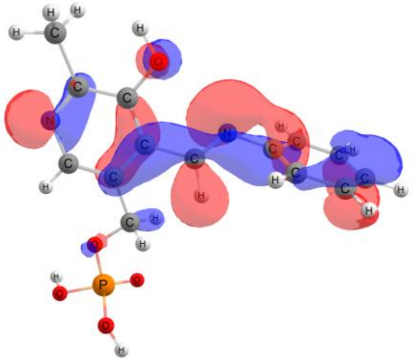 HOMO-5 → 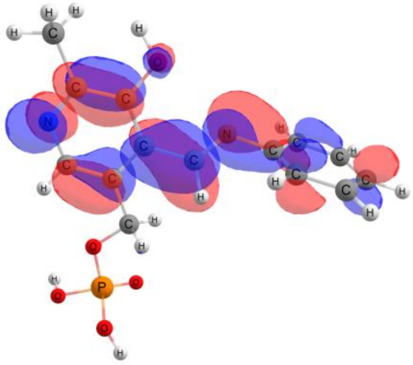 LUMO<br>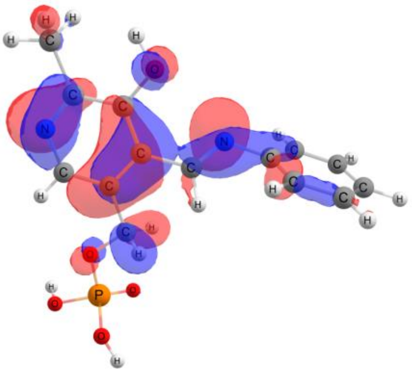 HOMO-4 ↗

2/1

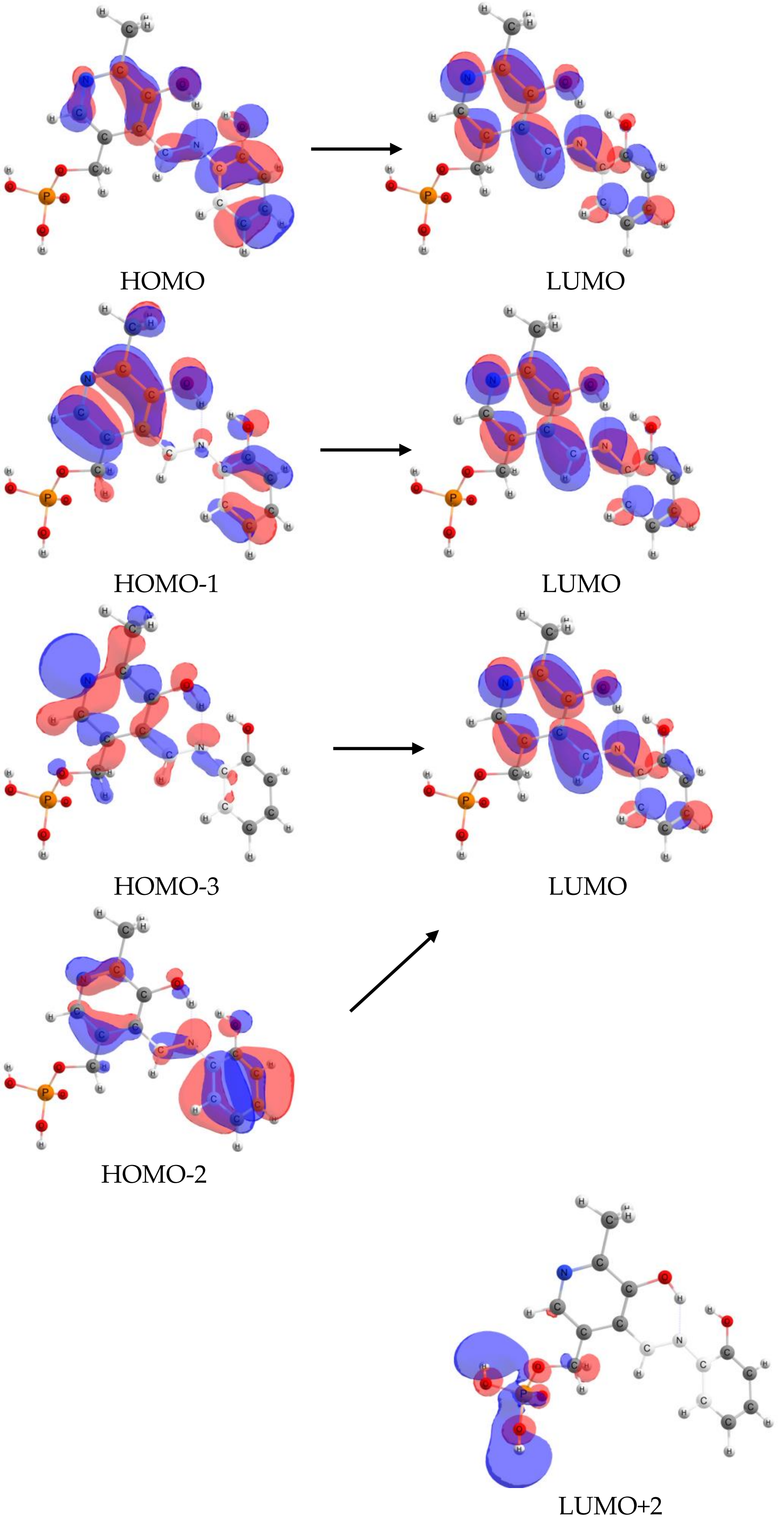

**2**/2	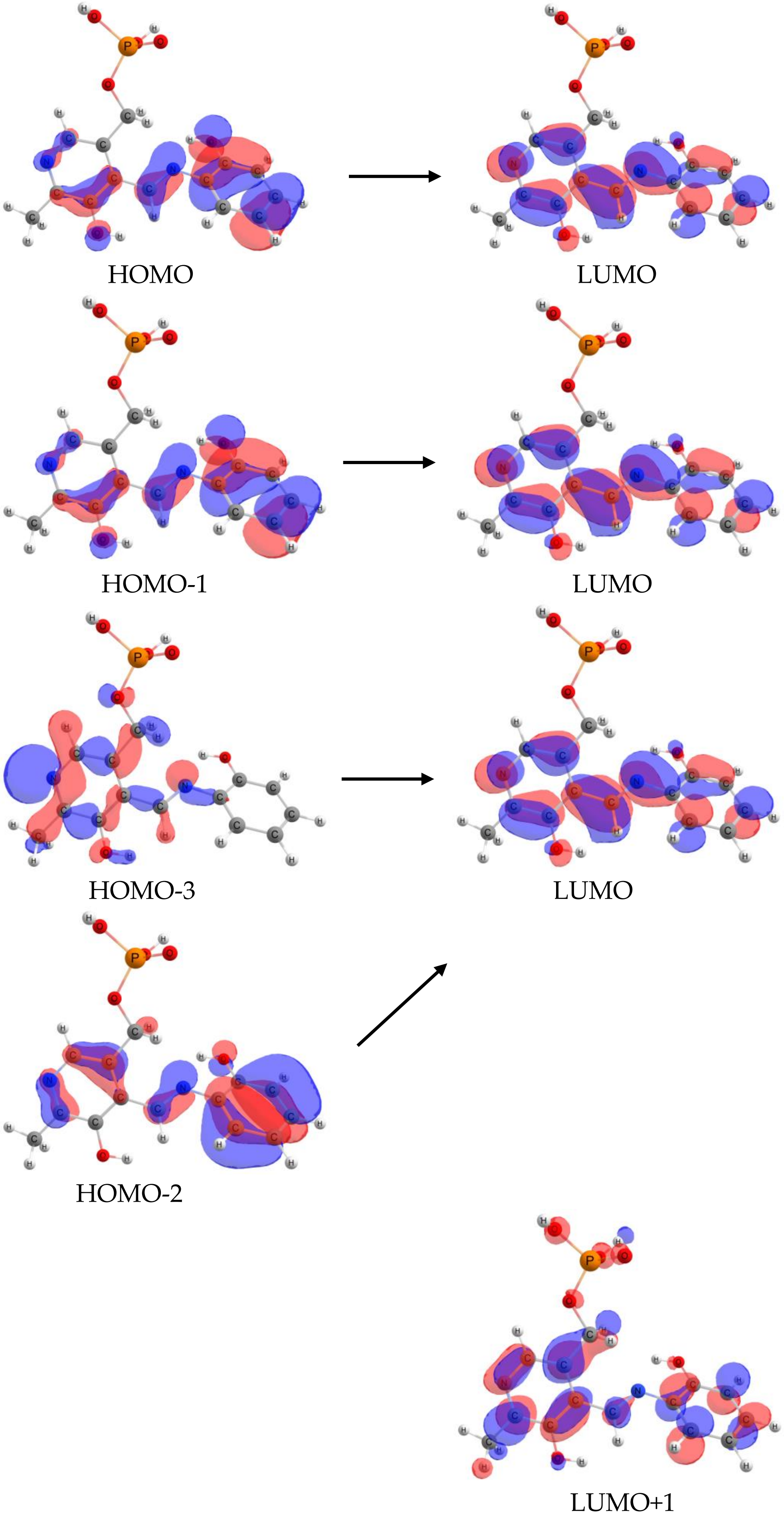

**3**/1	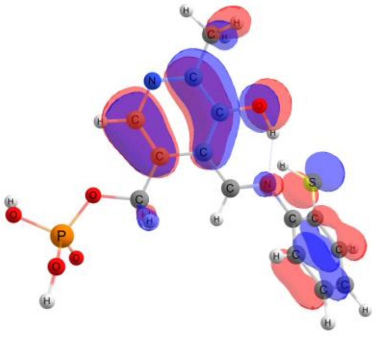<br>HOMO-1<br>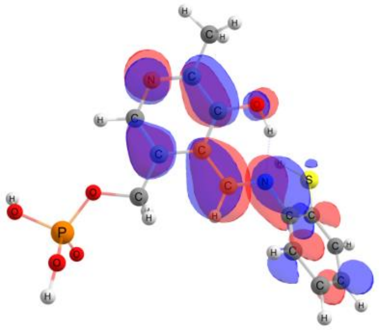<br>LUMO<br>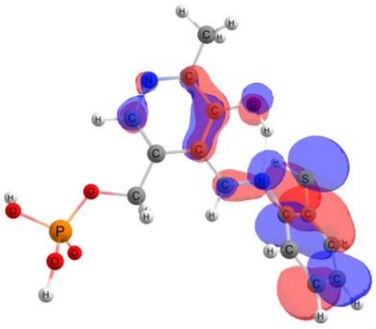<br>HOMO<br>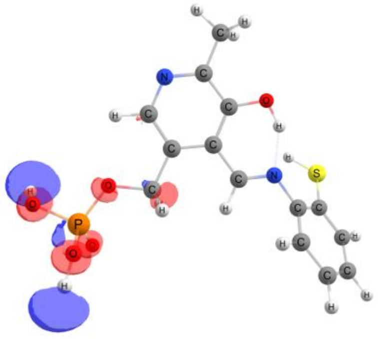<br>LUMO+1
**3**/2	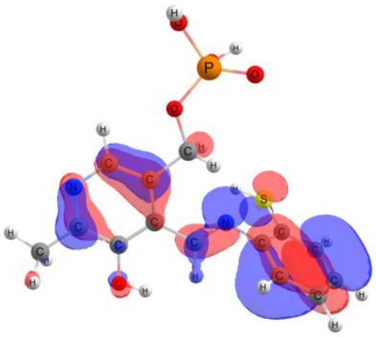<br>HOMO-2<br>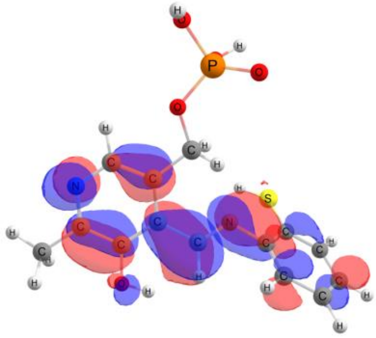<br>LUMO<br>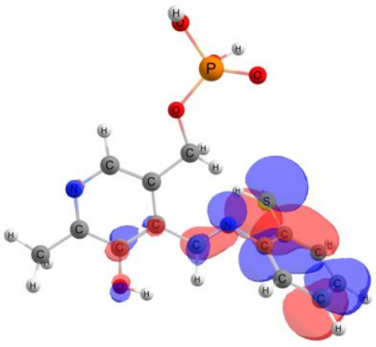<br>HOMO

	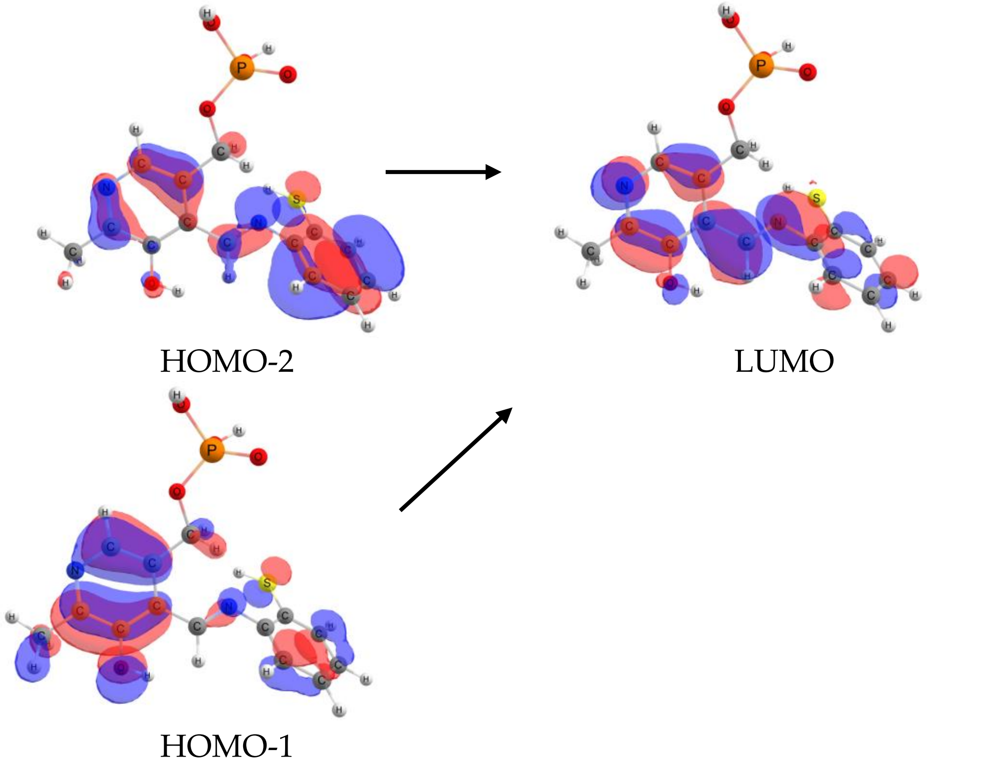 HOMO-2<br>LUMO<br>HOMO-1

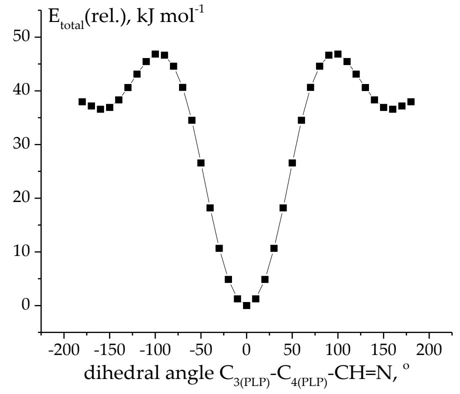

a

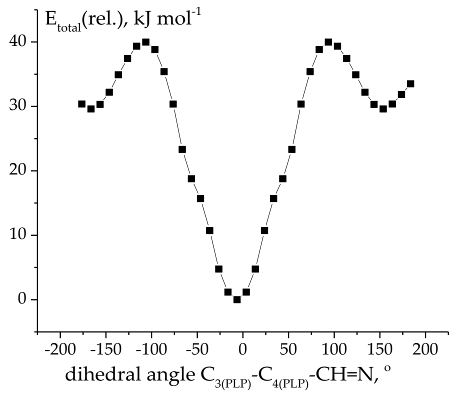

b

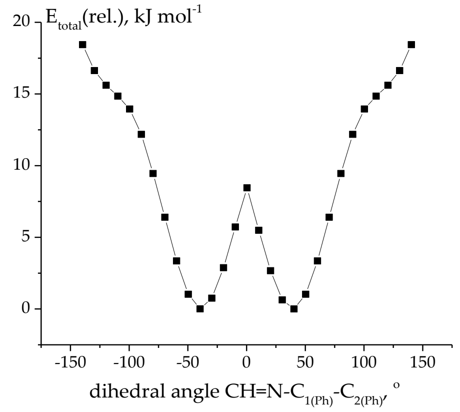

c

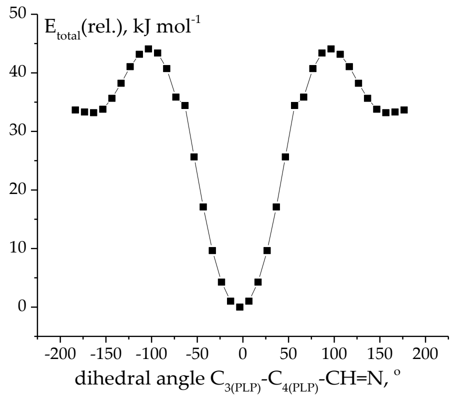

d

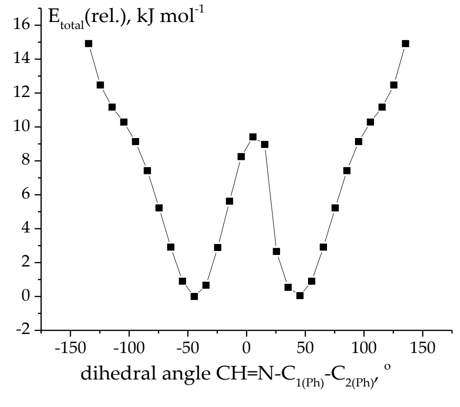

e

Figure S2. Potential energy surfaces drawn for the rotations of PLP (a, b, d) or phenyl (c, e) residue in SBs **1** (a), **2** (b, c), **3** (d, e)

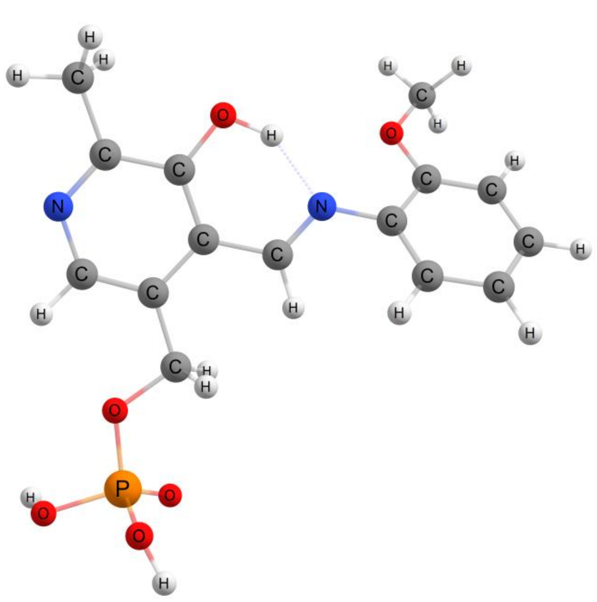

a

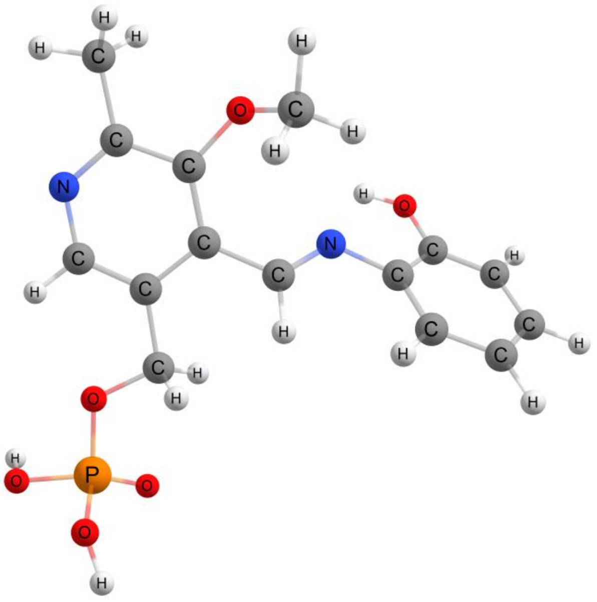

b

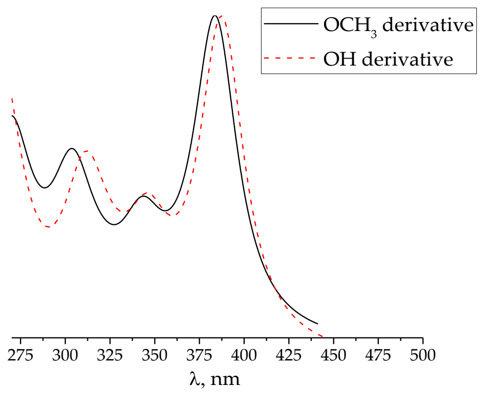

c

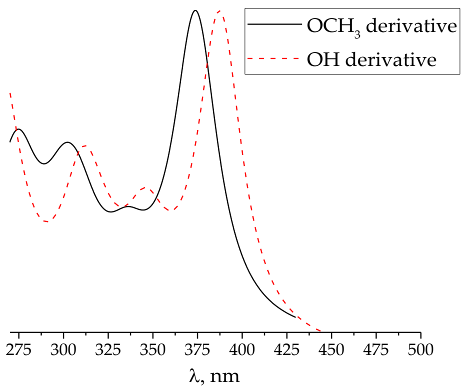

d

Figure S3. Optimized structure of SB **2** where OH group in position 2 of 2-hydroxyaniline (a) or position 3 of pyridoxal 5′-phosphate (b) are substituted by OCH_3 groups. Normalized TD DFT calculated spectra of the OCH_3 derivatives (black solid lines): spectrum (c) corresponds to the structure (a), while the spectrum (d) corresponds to the structure (b). The spectrum of SB **2** (conformer 1) is given for the comparison as red dashed lines

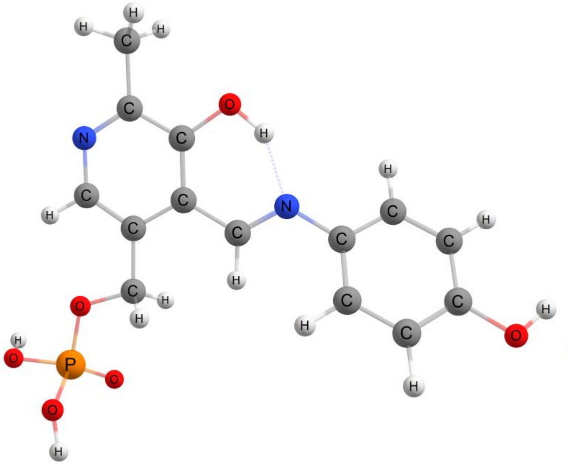

a

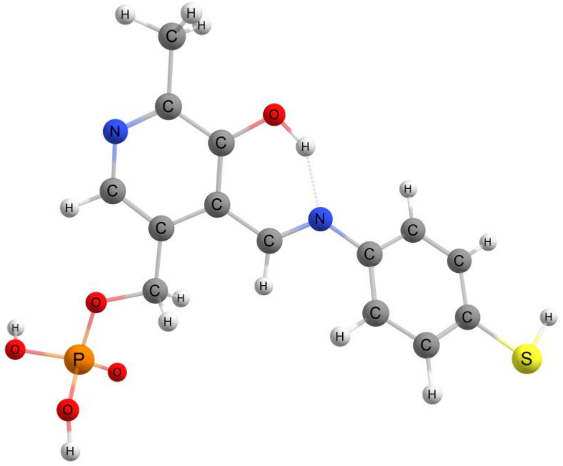

b

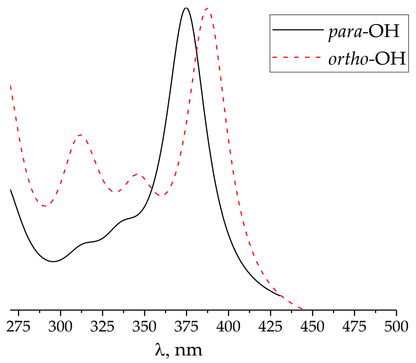

para-OH
ortho-OH
275 300 325 350 375 400 425 450 475 500
λ, nm

c

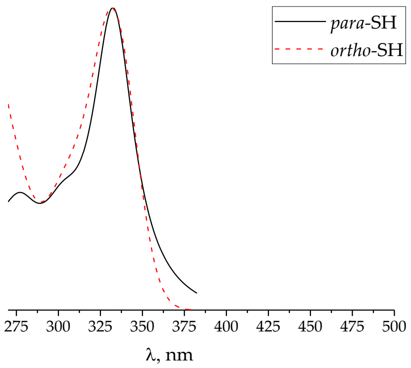

para-SH
ortho-SH
275 300 325 350 375 400 425 450 475 500
λ, nm

d

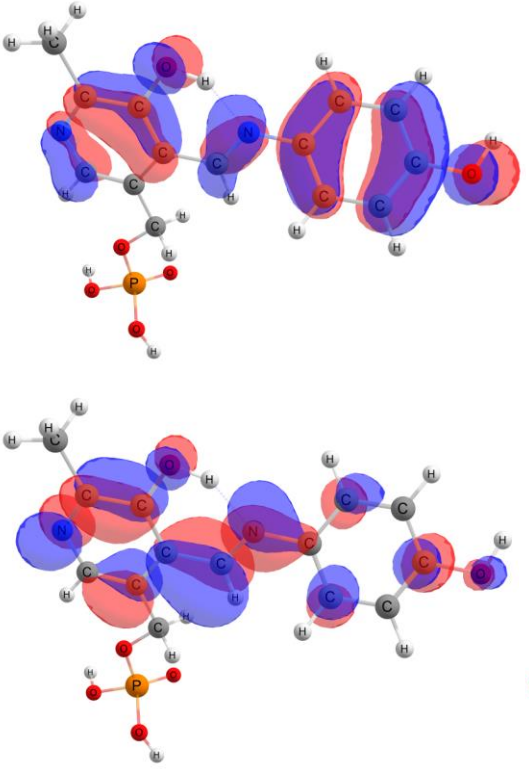

e

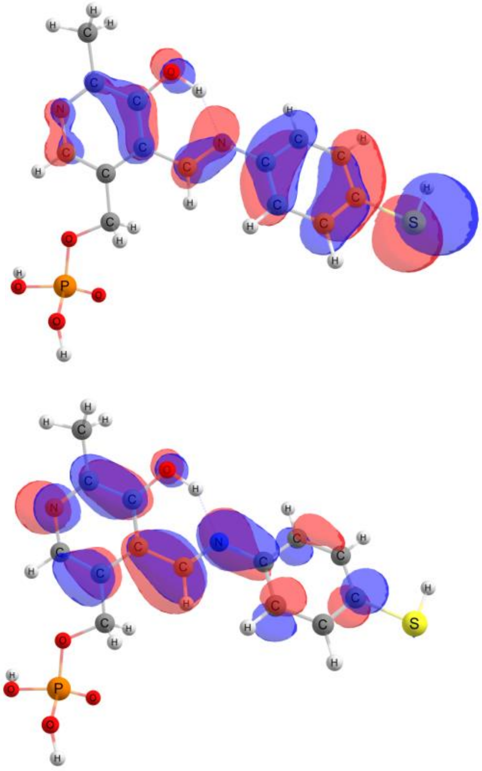

f

Figure S4. Optimized geometry of Schiff bases derived from pyridoxal 5′-phosphate and 4-hydroxyaniline (a), 4-mercaptoaniline (b). Normalized TD DFT calculated spectra of the OCH_3 derivatives (black solid lines): spectrum (c) corresponds to the structure (a), while the spectrum (d) corresponds to the structure (b). The spectra of SB **2** (conformer 1) and SB **3** (conformer 1) are given for the comparison as red dashed lines. HOMO and LUMO orbitals of 4-XH substituted Schiff bases: orbitals (e) correspond to the structure (a), while orbitals (f) correspond to the structure (b)

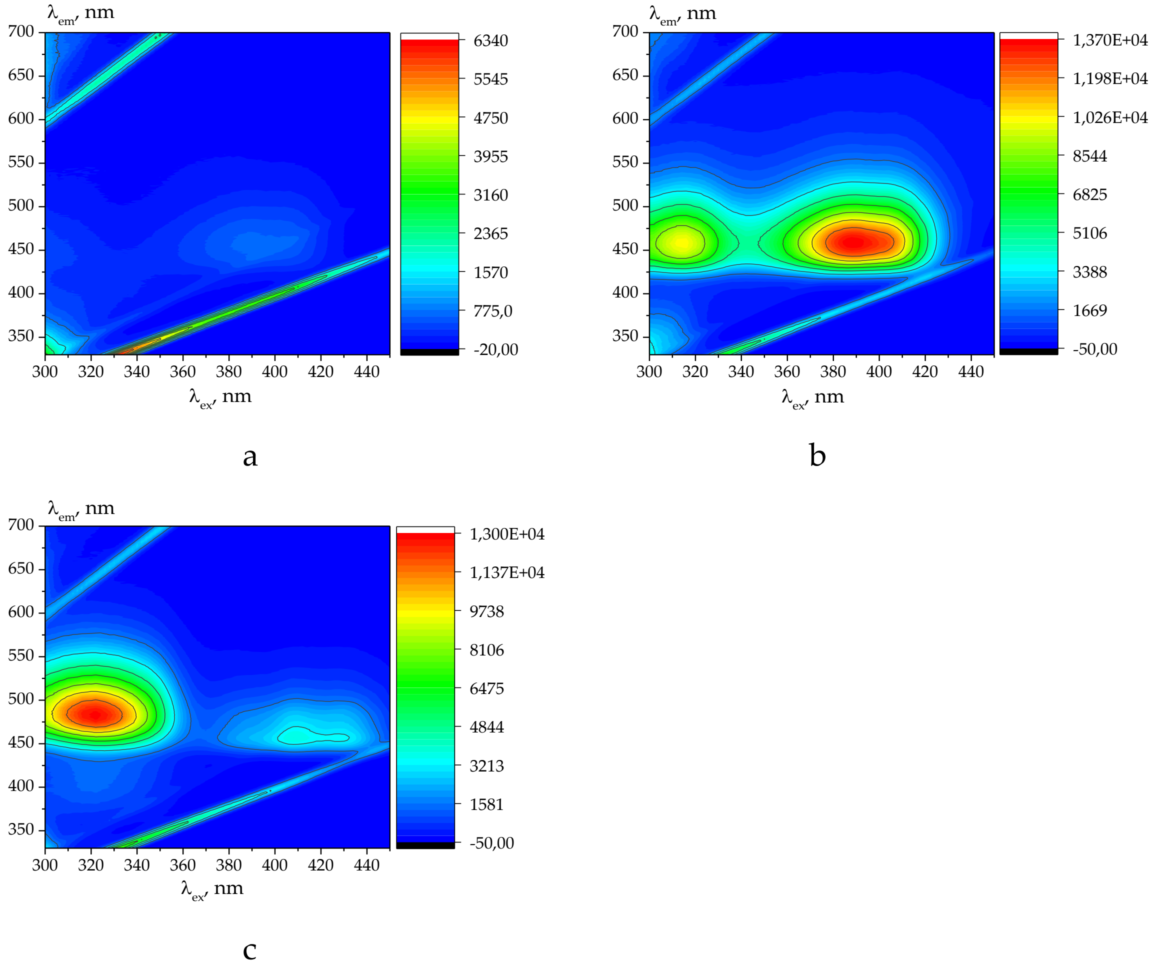

Figure S5. 2D fluorescent spectra (“heat map”) of the Schiff bases derived from pyridoxal 5′-phosphate and: a) aniline; b) 2-hydroxyaniline; c) 2-mercaptoaniline in DMSO. Concentration of Schiff base is 10^{-5} mol L^{-1}

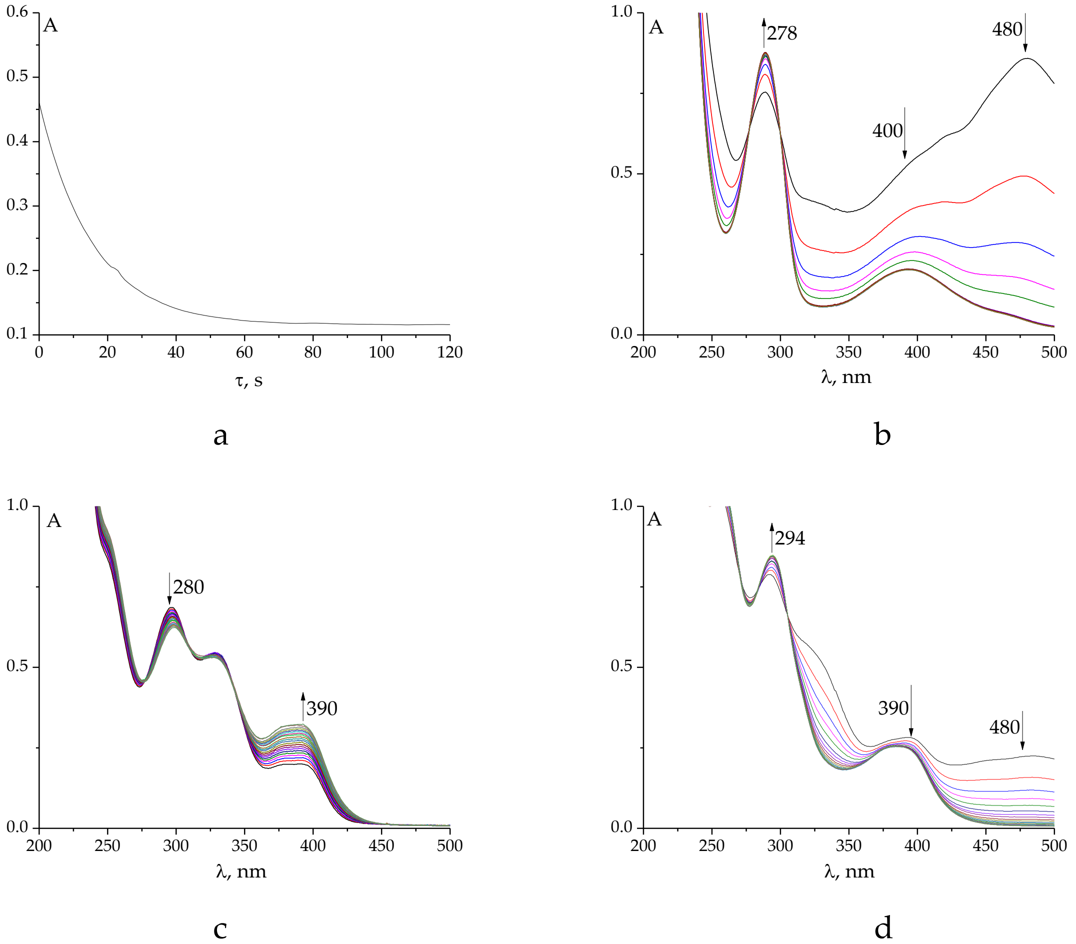

Figure S6. Changes in absorbance at $\lambda = 466$ nm of SB **2** (a) and evolution of UV-Vis spectra of SB **2** (b) and **3** (c, d) (10 to 20 spectra were registered with a delay of 45 s) after quick adding of SBs to aqueous buffer solution with pH of 7.0 (a, c) and 9.5 (b, d). Final concentration of SB in an aqueous medium is 10^{-4} mol L^{-1}

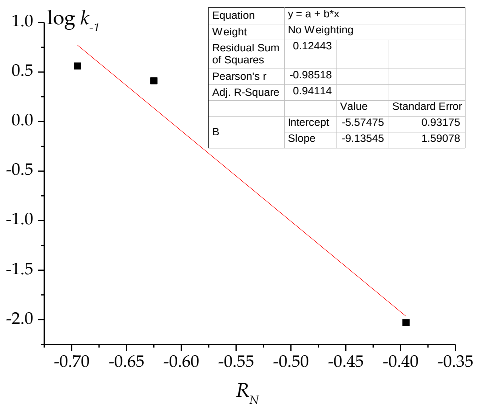

a

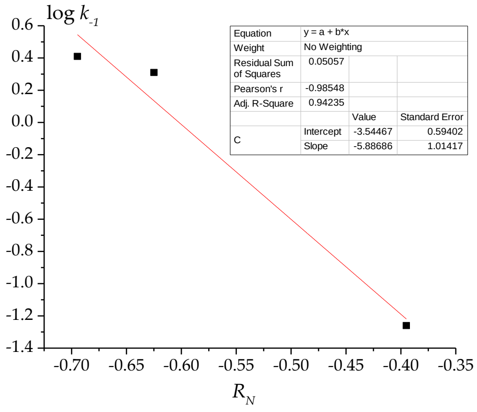

b

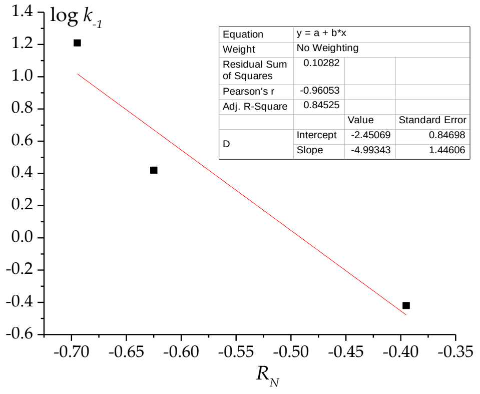

c

Figure S7. Correlation between rate constant of hydrolysis of Schiff base **1** (a), **2** (b), **3** (c) and a local reactivity difference index for imine nitrogen R_N

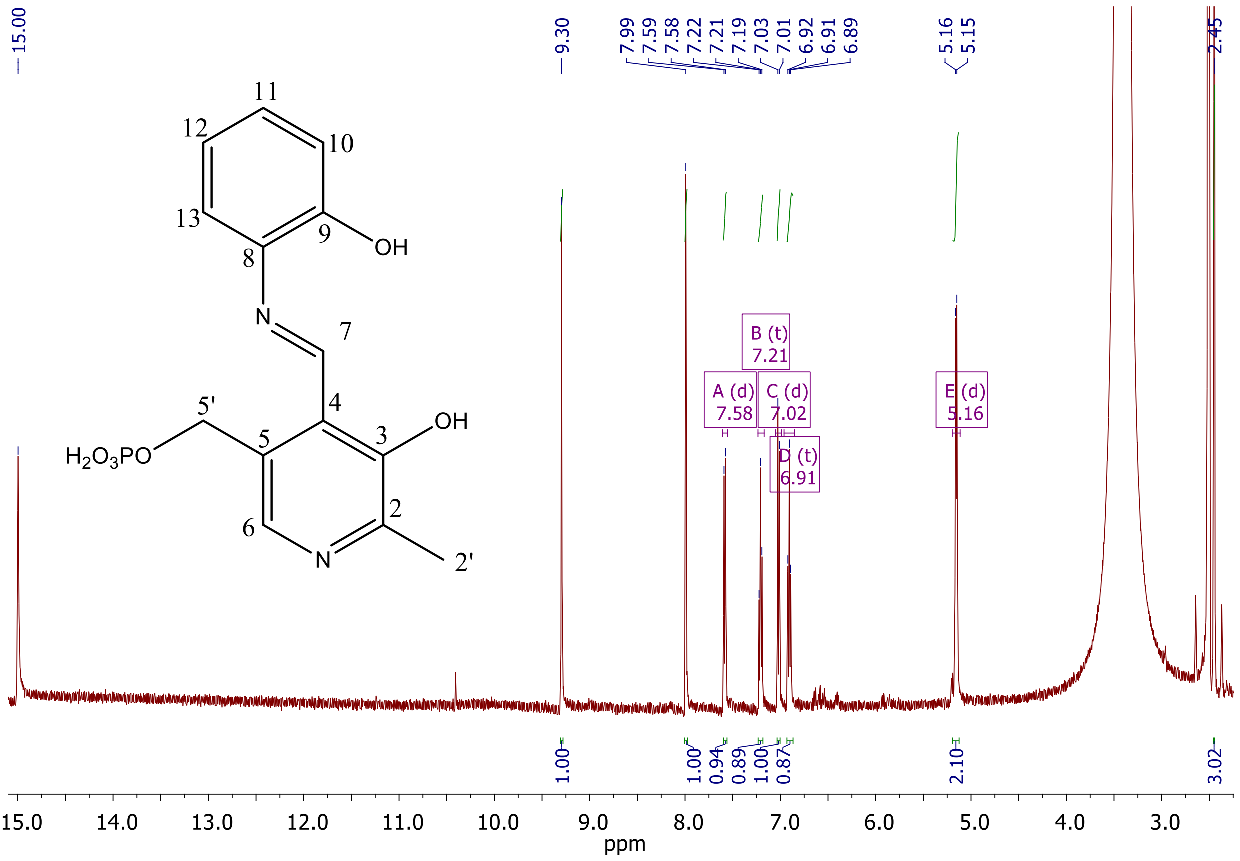
15.00
9.30
7.99 7.59 7.58 7.22 7.21 7.19 7.03 7.01 6.92 6.91 6.89
5.16 5.15
2.45
B (t) 7.21
A (d) 7.58
C (d) 7.02
D (t) 6.91
E (d) 5.16
1.00 1.00 0.94 0.89 1.00 0.87 2.10 3.02
H$_2$O$_3$PO
OH
N
ppm
15.0 14.0 13.0 12.0 11.0 10.0 9.0 8.0 7.0 6.0 5.0 4.0 3.0

a

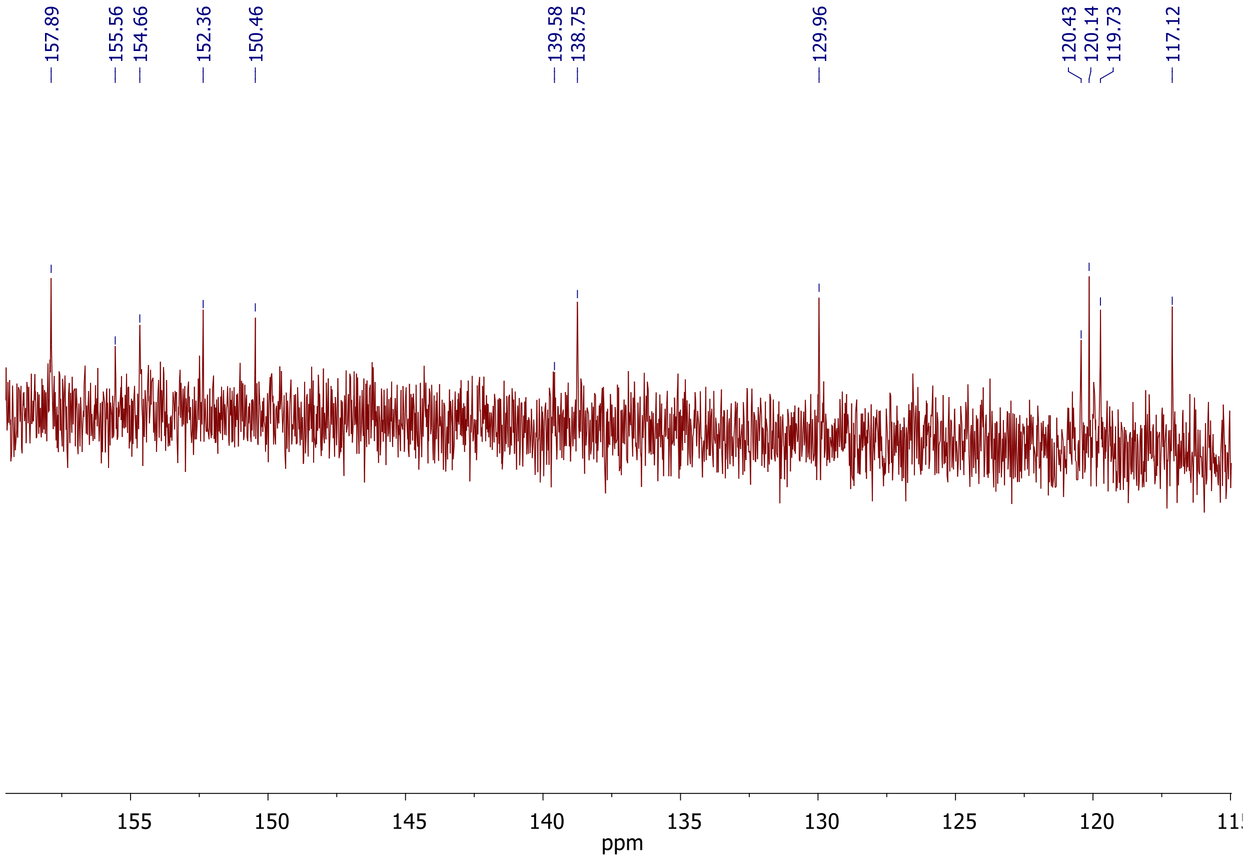
157.89
155.56
154.66
152.36
150.46
139.58
138.75
129.96
120.43
120.14
119.73
117.12
155 150 145 140 135 130 125 120
ppm

b

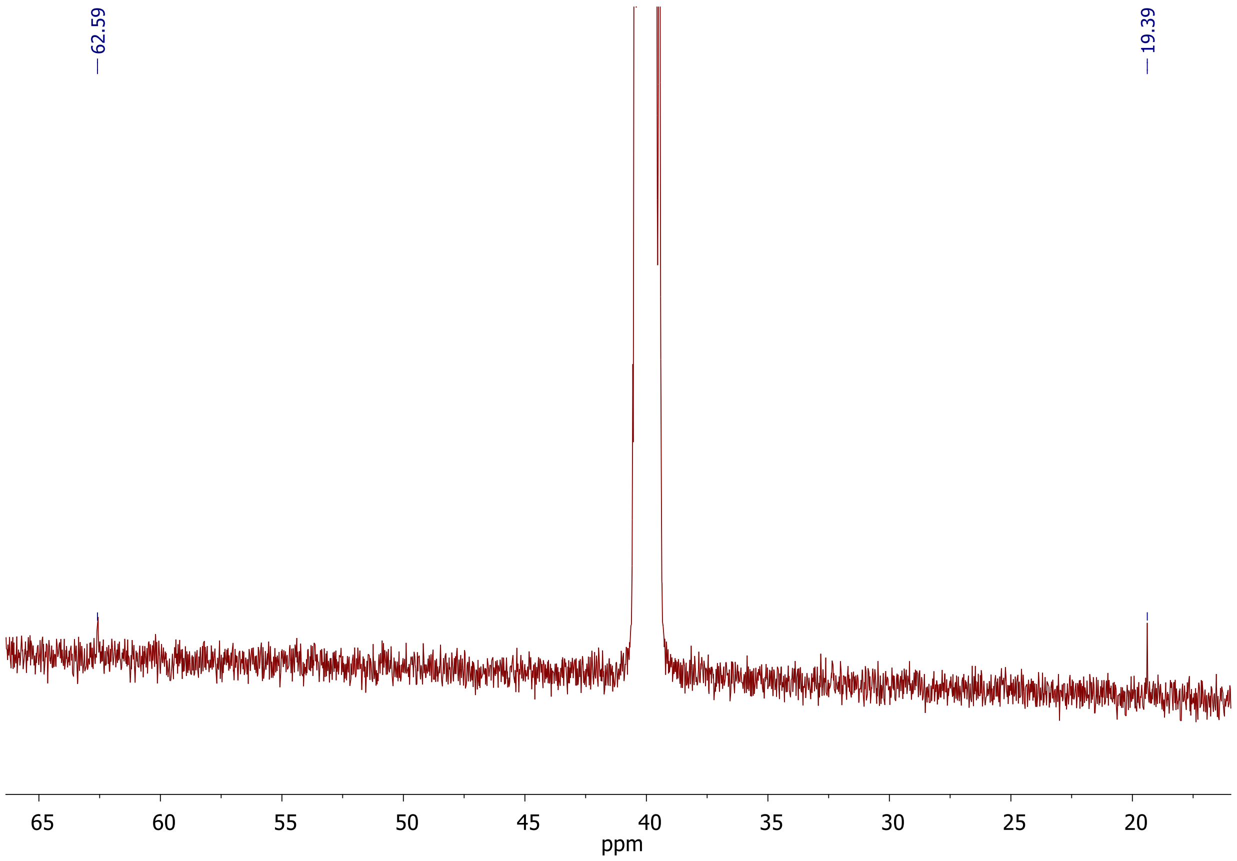

c

Figure S8. ^{1}H (a), ^{13}C (b, c) NMR spectra of the Schiff base derived from pyridoxal 5′-phosphate and 2-hydroxyaniline

The spectral assignment:

^{1}H NMR, δ, ppm (500.17 MHz, DMSO-d6): 15.00s (1H, phenolic OH), 9.30s (1H, H_7), 7.99s (1H, H_6), 7.58d (^{3}J = 7.4 Hz, 1H, H_{13}), 7.21t (^{3}J = 7.4 Hz, 1H, H_{11}), 7.02d (^{3}J = 7.4 Hz, 1H, H_{10}), 6.91t (^{3}J = 7.4 Hz, 1H, H_{12}), 5.16d (^{3}J = 6.5 Hz, 2H, $H_{5'}$), 2.45s (3H, $H_{2'}$).

^{13}C NMR, δ, ppm (125.77 MHz, DMSO-d6): 158.9 (C_3), 155.6 (C_7), 154.7 (C_9), 152.4 (C_2), 150.5 (C_6), 139.6 (C_8), 138.8 (C_4), 130.0 (C_{11}), 120.4 (C_5), 120.1 (C_{12}), 119.7 (C_{13}), 117.1 (C_{11}), 62.6 ($C_{5'}$), 19.4 ($C_{2'}$).

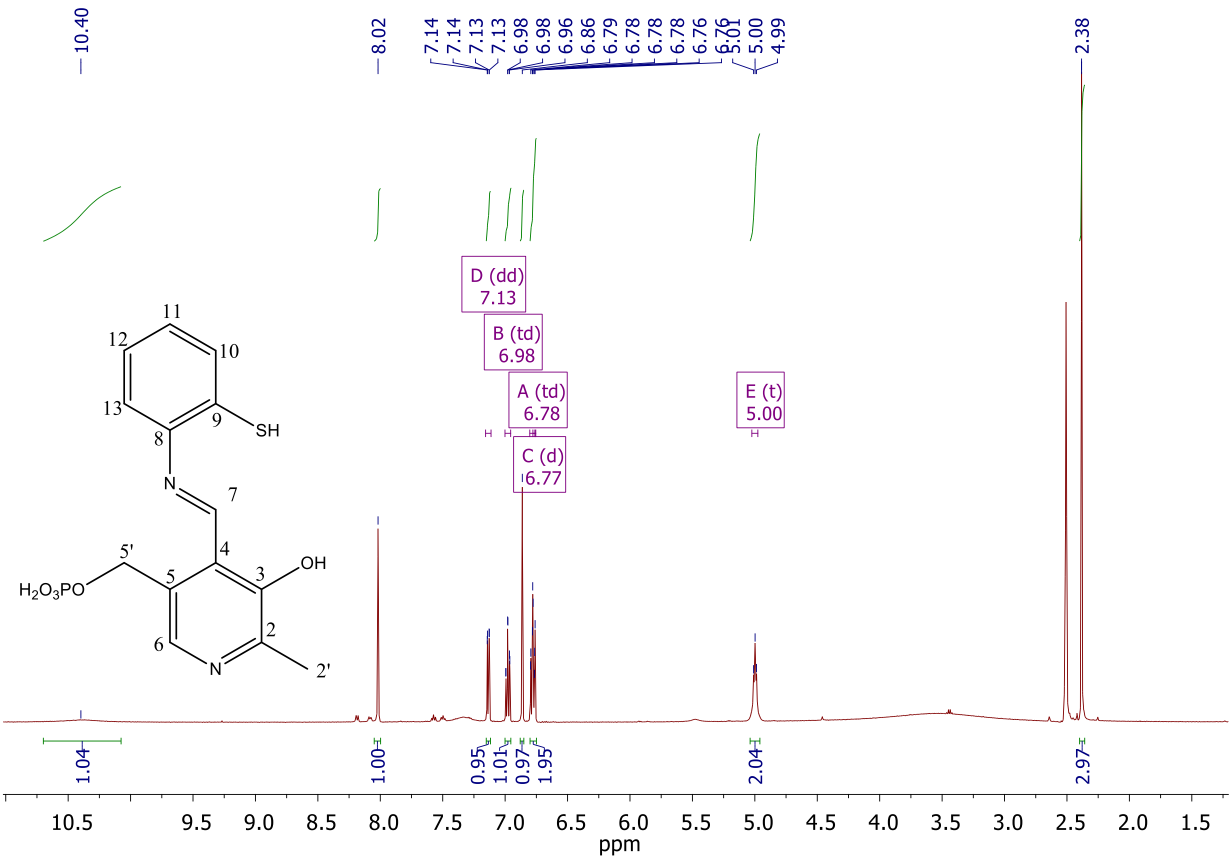

Figure S9. ^{1}H NMR spectrum of the Schiff base derived from pyridoxal 5′-phosphate and 2-mercaptoaniline

The spectral assignment:

^{1}H NMR, δ, ppm (500.17 MHz, DMSO-d6): 10.40s (1H, SH), 8.02s (1H, H_7), 7.13dd (^3J = 7.5 Hz, ^4J = 1.0 Hz, 1H, H_{10}), 6.98td (^3J = 7.5 Hz, ^4J = 1.0 Hz, 1H, H_{11}), 6.78td (^3J = 7.5 Hz, ^4J = 1.0 Hz, 1H, H_{12}), 6.77d (^3J = 7.5 Hz, 1H, H_{13}), 5.00t (^3J = 5.9 Hz, 2H, $H_{5'}$), 2.38s (3H, $H_{2'}$).
